# Experience of living with psoriasis in Brazil: a Global Psoriasis Atlas online survey

**DOI:** 10.1111/ijd.17387

**Published:** 2024-07-17

**Authors:** Jaquelini Barboza da Silva, Alison K. Wright, André V. E. Carvalho, Christopher E. M. Griffiths, Darren M. Ashcroft

**Affiliations:** ^1^ Department of Life Sciences University of Santa Cruz do Sul Santa Cruz do Sul Brazil; ^2^ Centre for Pharmacoepidemiology and Drug Safety, Division of Pharmacy and Optometry School of Health Sciences, Faculty of Biology, Medicine and Health, NIHR Manchester Biomedical Research Centre, University of Manchester Manchester UK; ^3^ Psoriasis Outpatient Clinic, Hospital Moinhos de Vento Porto Alegre Brazil; ^4^ Centre for Dermatology Research, NIHR Manchester Biomedical Research Centre, Manchester Academic Health Science Centre University of Manchester Manchester UK; ^5^ Department of Dermatology King's College Hospital, King's College London London UK

**Keywords:** psoriasis, quality of life, social stigma, access to care, inflammatory diseases

## Abstract

**Background:**

Psoriasis significantly burdens patients' lives, but there is limited data on this in Brazil.

**Methods:**

Between May 2022 and January 2023, we conducted a cross‐sectional online survey of 563 Brazilian residents aged ≥18 years who had been diagnosed with psoriasis. Spearman's correlation (*r*) was used to test the correlation between self‐assessed disease severity (Simplified Psoriasis Index [saSPI] extent score; range 0 [clear/minor] to 40 [widespread/severe]) and health‐related quality of life (QoL, score of 1 means perfect health) and capability (ICECAP‐A: score of 1 means full capability) measures. Multivariable linear regression was used to identify predictors of QoL and capability. A thematic analysis examined the free‐text responses and identified common themes.

**Results:**

The mean age of participants was 42.1 ± 12.4 years, and over half had at least one other long‐term condition. The mean QoL score was 0.59 ± 0.25, and the mean capability score was 0.71 ± 0.21. At the time of survey completion, over 80% of respondents reported some level of pain and/or discomfort, and 86% reported feeling anxious and/or depressed. The mean self‐assessed saSPI was 7.8 ± 8.6, which negatively correlated with health‐related QoL (*r* = −0.49, *P* < 0.05) and capability (*r* = −0.44, *P* < 0.05). Significant predictors of poorer QoL and reduced capability included high saSPI, number of psoriasis flares and comorbidities, female gender, Black ethnicity, and employment status (unemployed, long‐term sick). Frequently reported areas that impacted patients were social stigma/prejudice, powerlessness, lack of education and public awareness, and difficulty obtaining appropriate care/treatment.

**Conclusions:**

We found that the clinical manifestations, severity, and associated comorbidities of psoriasis negatively impacted health‐related QoL and capability, along with feelings of stigmatization and barriers to specialist treatment. This highlights the need for better access to care and awareness of the disease to improve the lives of people living with psoriasis in Brazil.

## Introduction

Psoriasis is a chronic, immune‐mediated skin disease predominantly characterized by remission and exacerbation.^1^ It is associated with multiple comorbidities and increased mortality. It can have a considerable impact on quality of life (QoL), with many patients experiencing a range of difficulties, including pain, discomfort, physical disability, impairment of daily activities; social stigmatization; psychological distress (low self‐esteem, stress); substance‐related disorders; and mental health disorders including anxiety, depression, and suicidal ideation.^2^, ^3^ Due to the nature of the disease, life‐long treatment may be required, which can place a significant economic burden on patients and the healthcare system.^4^

The Global Psoriasis Atlas (GPA) previously estimated worldwide regional prevalence rates of psoriasis^5^; prevalence varied from 0.14% (95% CI: 0.05–0.40%) in East Asia to 2.36% (1.10–5.04%) in Scandinavia (Norway). There is a paucity of data on the epidemiology of psoriasis in Brazil; however, a geographical telephone survey of Brazilian state capitals identified residents diagnosed with psoriasis and estimated prevalence across five regions of Brazil (South, Southeast, Central‐West, North, and Northeast).^6^ In a population of 8,947 residents, the overall prevalence of psoriasis was 1.31% (1.10–1.51%), with the lowest rate reported in the north (0.92% [0.80–1.33%]) and highest in the southeast (1.88% [1.30–2.46%]).^6^ The variability in prevalence across the country was associated with the differing ethnic composition of the regions (predominantly Indigenous peoples of the Americas in the north and Europeans in the south and southeast), climate, dermatological services, and private health insurance coverage (highest in the south and southeast regions).^6^

The healthcare system in Brazil comprises public, private, or supplemental healthcare, with approximately 75% of residents relying solely on public services.^7^ For moderate to severe psoriasis, biologics have shown high efficacy and superiority over conventional treatments; however, they are considerably more expensive.^8^, ^9^ Biologics have been available in Brazil since 2006 but have only been subsidized by the government since 2019 (2021 for private healthcare providers) for patients whose treatment with conventional drugs was unsuccessful or contraindicated. Maul *et al*.^10^ examined the availability of systemic treatments in 2020 and found that only 37% of patients with severe psoriasis received biologics. The pricing model of biologics in Brazil has created distortions, making the drugs more expensive than in other countries and limiting access to these drugs.^11^

In 2022, we conducted an online survey of people living with psoriasis to better understand the burden of psoriasis on social and physical activities, capability, quality of life, satisfaction with available treatments, and whether the impact of living with psoriasis on health and capability was associated with patient characteristics, severity of psoriasis, and/or type of psoriasis treatment used.

## Materials and methods

### Patient recruitment and survey

To identify individuals with psoriasis, a convenience approach was taken. Participants were recruited through email from patient associations and social media platforms (Facebook, YouTube, and Instagram). The survey included screening questions to determine if potential participants qualified for inclusion, and consent to participate was sought. Eligible individuals were Brazilian residents aged ≥18 years with literacy proficiency, who had been diagnosed with psoriasis. The Santa Cruz University Ethical Committee approved this study with the Certificate of Presentation of Ethical Application number 50692221.0.0000.5343.

An online structured questionnaire developed by Ng'ambi^12^ was translated from English into Portuguese using the translation by committee and back‐translation approaches.^13^ JBdS and AVEC worked separately to generate a translated version, and a consensus questionnaire was produced. The Portuguese questionnaire was translated back into the source language (English) by an independent translator blinded to the original questionnaire. The two source language versions were then compared. The questionnaire collected data on demographics, psoriasis severity, psoriasis medications, comorbidities, health‐related measures EQ‐5D‐5L (EuroQoL 5‐Dimension 5‐Level) quality of life health status, and ICECAP‐A (ICEpop CAPability measure for Adults) capability, and free‐text responses from participants providing an insight into their experiences of living with psoriasis. The EQ‐5D‐5L responses were expressed as one of five severity levels for each of the five dimensions (mobility, self‐care, usual activity, pain and/or discomfort, anxiety and/or depression) and aggregated to generate a single score where 1 represents “perfect” health (full functional quality of life), zero represents “death” and below zero represents ‘worse than death’.^14^ Additional information on the construction of the score is provided in Data S1. ICECAP‐A measures the level of capability for each of the attributes (stability, attachment, independence, achievement, and enjoyment), generating an aggregated single score for each individual that ranges between 0 (no capability) and 1 (full capability). Psoriasis severity was defined using the aggregated single self‐assessment Simplified Psoriasis Index (saSPI) severity score (range 0 to 40) comprising the extent score (summed score across each body area recorded as 0 [clear], 0.5 [obvious], or 1 [widespread]) and the average plaque severity score for their condition (0 [clear or slight redness], 1 [mild redness or scaling], 2 [definite redness], 3 [moderately severe], or 4 [very red and inflamed]).^15^

$$\text{saSPI}=\left(\Sigma \;\text{body area extent scores}\right)\times \text{(average plaque severity)}$$

Further details on the questionnaire content are provided in Data S1.

### Statistical analyses

Data were described as the frequency and percentage for categorical data and mean ± standard deviation (SD) for continuous data. The Spearman correlation coefficient, Rho (ρ), was used to test the correlation between the individual areas of the body affected, severity extent score, average plaque severity score, and the self‐assessed saSPI score with the outcomes, health‐related quality of life, and capability. Linear regression models explored the impact of the saSPI score and patient characteristics on health‐related quality of life and capability. The beta value from the regression is the standardized coefficient and was used to rank the importance of each predictor variable. All statistical analyses were performed using Stata, version 16 (StataCorp LLC, College Station, TX, USA).

A thematic analysis was conducted to examine the free‐text responses from participants describing their experiences of living with psoriasis and identify common themes. This involved a six‐step process^16^: familiarization with the data, generating initial codes, searching for themes, reviewing themes, defining and naming themes, and producing the final analysis. The data were coded and categorized by JBdS. An inductive, data‐driven approach to theme identification was employed within a constructivist epistemology framework. The wider research team reviewed the themes (DMA, AKW, CEMG, AVEC) and revised them to combine common themes.

## Results

A total of 563 people participated in the online survey. Their ages ranged from 18 to 80 years, with a mean of 42.1 ± 12.4 years. Females comprised 73.5% of respondents, 67.5% identified as White, 25.9% mixed, 5.3% Black, 0.9% Asian, and 0.4% other. Detailed demographic and clinical characteristics of the respondents are shown in Table S1. Most participants had psoriasis duration >10 years (66.8%), were non‐smokers (87.9%), but consumed alcohol at least once a week or more (53.1%). In the past year, 46% of respondents experienced one flare, and 27% experienced multiple flares. The psoriasis medications used are detailed in Appendix S2. While 83% of respondents received prescribed medications (topicals, injectables, and oral) or used other treatments, including UV/sun exposure, diet modification, and alternative treatments, less than half of respondents were satisfied. Comorbidities such as mental health conditions (anxiety and depression), arthritis or back or joint problems, hypertension, kidney or liver disease, and type 2 diabetes were prevalent, with 52.4% of respondents reporting at least one other long‐term condition (Table S1).

**Figure 1:**
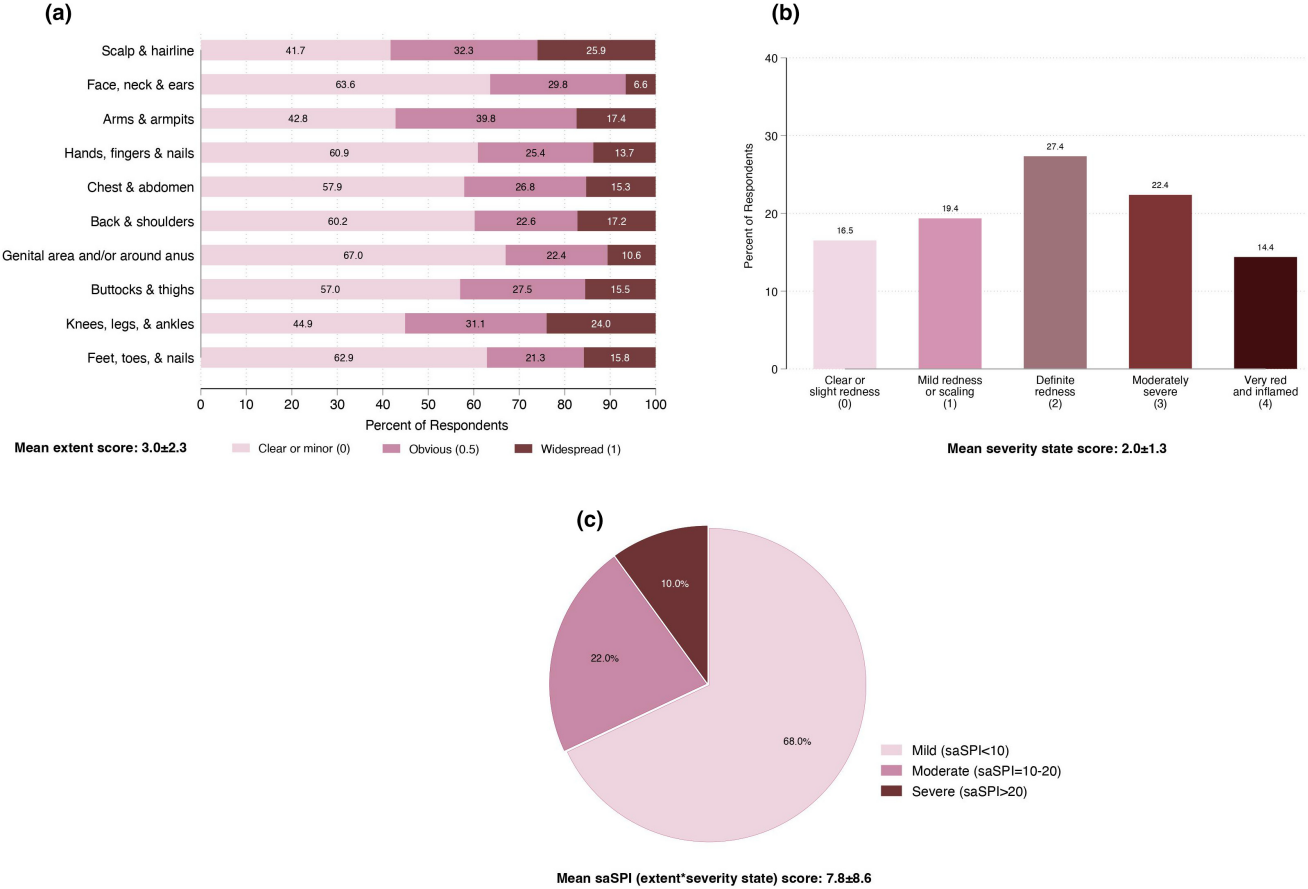
Self‐assessment of the extent of psoriasis (a), overall state of severity (b), and Simplified Psoriasis Index (saSPI) severity score (c)

Figure 1 shows the frequency distribution of responses for the extent of psoriasis affecting ten body areas (Panel a), the overall state and severity of psoriasis (Panel b), and the grades of saSPI (Panel c). The mean saSPI severity score was 7.8 ± 8.6 with most patients (68%) having mild psoriasis (saSPI < 10), 22% with moderate psoriasis (saSPI: 10–20), and 10% with severe psoriasis (saSPI > 20). Moderate or widespread involvement of the scalp and hairline was the most frequently reported area of the body affected (58.2%), followed by the arms and axillae (57.2%), knees, legs, and ankles (55.1%), buttocks and thighs (43.0%), and chest and abdomen (42.1%).

**Figure 2:**
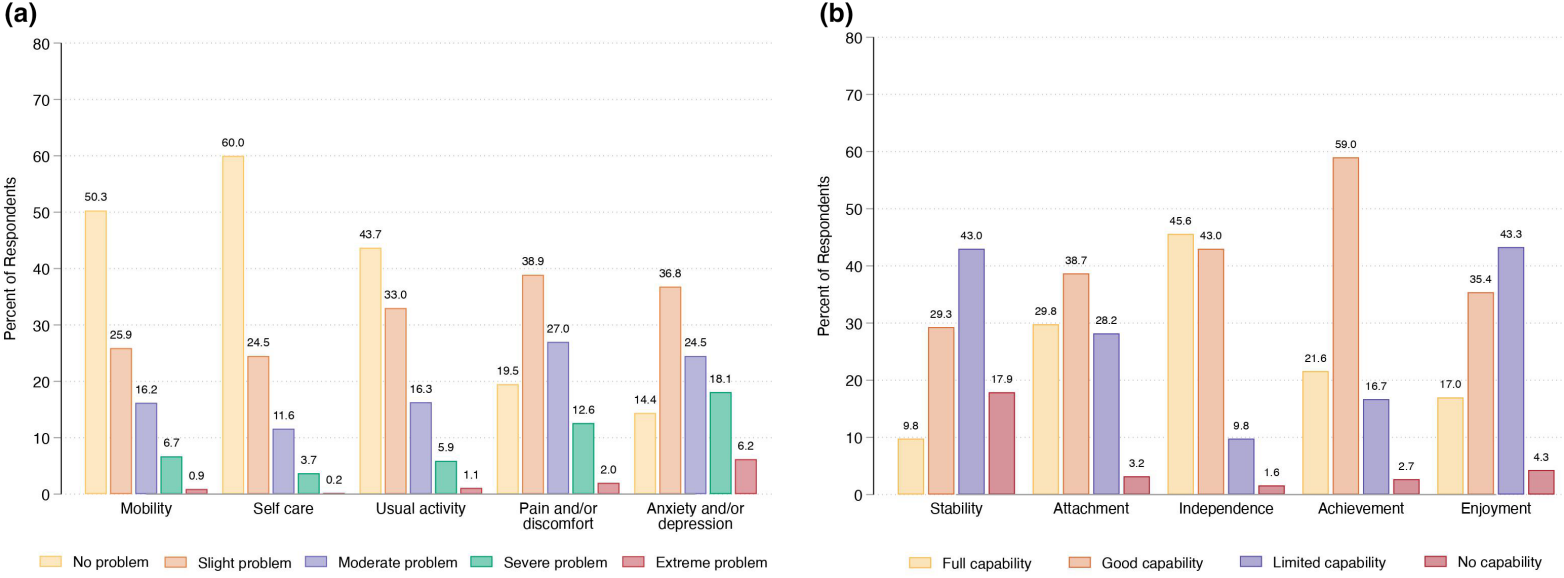
Patient responses to the quality‐of‐life measures (a) and capability measures (b)

Figure 2 shows the reported levels for each dimension of the QoL score (Panel a) and the capability score (Panel b). The mean health‐related QoL score was 0.59 ± 0.25. At the time of survey completion, most respondents did not report any issues with mobility, self‐care, or usual activities; however, over 80% of respondents reported some level of pain and discomfort, and 86% had anxiety and depression. The mean capability score, representing the impact of psoriasis on well‐being, was 0.71 ± 0.21, with the attributes related to stability, enjoyment, and attachment most affected by psoriasis.

**Figure 3:**
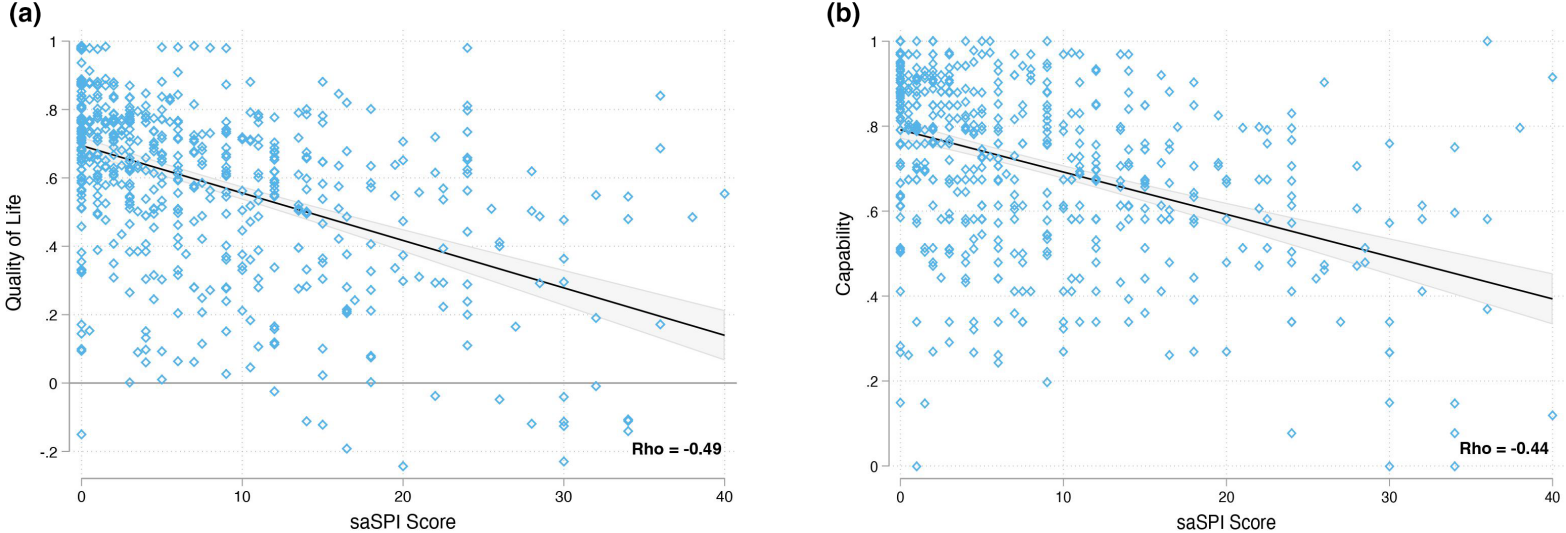
Correlation between self‐assessment Simplified Psoriasis Index (saSPI) severity and health‐related quality of life (a) and capability (b)

The Spearman correlation coefficient (*r*) was used to examine the association between severity and health‐related quality of life and capability. There were significant negative correlations observed between each individual area of the body affected, extent score, overall state of psoriasis score, and saSPI severity score with the health outcomes (quality of life and capability); Appendix S3 and Figure 3. As the severity scores increased, quality of life and capability decreased; *r* = −0.49 (*P* < 0.05) and *r* = −0.44 (*P* < 0.05), respectively. Linear regression models were used to estimate the association between several patient characteristics and saSPI on health‐related quality of life and capability; Appendix S4. Significant predictors of poorer quality of life included increased saSPI, female gender, Black ethnicity, unemployment or being off work due to long‐term illness, number of comorbidities, number of psoriasis flares, and use of injectable or alternative treatments. saSPI, comorbidities, flares, and gender were found to be the strongest predictors of lower quality of life. Significant predictors of reduced capability included increased saSPI, female gender, Black ethnicity, lower educational attainment, unemployment or off work due to long‐term illness, the number of comorbidities, and the number of psoriasis flares. saSPI, comorbidities, employment status, educational attainment, flares, and gender were the strongest predictors of reduced capability. Exposure to sunlight was a significant predictor of improvements in capability.

In the thematic analysis of the respondents' free‐text responses describing their experience of living with psoriasis, four main themes were identified: (i) stigmatization and prejudice; (ii) powerlessness; (iii) lack of public awareness; and (iv) difficulty obtaining appropriate specialist treatment. Many respondents reported feelings of stigma, prejudice, and judgment, which impacted their relationships, work, self‐esteem, and confidence. This was compounded by a lack of public awareness of psoriasis and a misbelief that psoriasis was contagious or was leprosy. There were also feelings of powerlessness to the condition, even during remission. The impact of this on their lives was multifaceted, resulting in discomfort, insecurity, and worsening mental health and wellbeing. Barriers to accessing specialist dermatological treatments, particularly biologic therapies, were another major challenge, with respondents reporting difficulty obtaining appropriate and effective treatments. A selection of these free‐text responses from respondents is provided in Appendix S1.

## Discussion

The results of this survey provide essential insights into the experience of living with psoriasis in Brazil. We found that the clinical extent of psoriasis, the state of psoriasis, and the overall severity of psoriasis negatively affected patients' quality of life (QoL) and capability. Over half of the psoriasis population had at least one other long‐term condition, with a high proportion reporting pain or discomfort and anxiety or depression. The challenges participants faced included social stigma and prejudice, poor social awareness of psoriasis, issues with access to appropriate specialist care and treatment, and treatment dissatisfaction. These findings highlight the need for more comprehensive and effective patient‐centered care and public awareness of the disease to improve the lives of people living with psoriasis in Brazil.

In the surveyed population, patients reported barriers to accessing dermatologists and adequate treatment for their psoriasis. Health and social inequalities persist in Brazil^17^, ^18^; having a higher income is associated with greater utilization of healthcare, and those with private insurance often seek care more often than those who do not have private insurance and rely solely on public services.^17^, ^19^ Access to dermatologists in Brazil can be limited due to private insurance scarcity in urban, underserved communities. Patients seeking an appointment through the public health system can face long waiting times for face‐to‐face evaluations.^20^ This can make it challenging to obtain a timely diagnosis and treatment.

In the treatment of moderate‐to‐severe psoriasis, conventional systemic treatments and phototherapy are the first‐line treatments, with biologics considered second‐line (when first‐line has failed, is contraindicated, or has adverse side effects) due to their superiority in terms of efficacy and safety compared to conventional systemic therapies.^7^, ^9^, ^10^ It has previously been reported that there is a low availability of phototherapy in public and private health systems in Brazil, even though this is a highly efficacious therapy that facilitates long‐term psoriasis control and could reduce the need for more costly treatments.^7^ While phototherapy is generally a lower‐cost therapy compared to biologics in many countries, it is still prohibitive in Brazil due to the high costs of the equipment, its running costs, and maintenance, along with limited facilities offering this treatment and high transportation costs for patients traveling to these clinics.^7^, ^21^ Access to biologics in developing countries varies greatly, and patients have reported difficulties in drug availability and obtaining prescribed medications.^18^, ^22^ In Brazil, biologics have been available since 2006; however, these drugs have only been subsidized by the government for patients who have failed with systemic conventional medicines since 2019 in the public healthcare system and since 2021 by private healthcare providers. While some biologics may now be prescribed for moderate‐to‐severe psoriasis, access depends on each state, and factors such as the complex administrative process involved and distorted pricing (related to the high tax burden, fluctuating US dollar exchange rate, and lack of monitoring and revision of prices) may still impede prompt availability.^11^, ^23^

Anxiety and depression are commonly reported in people with psoriasis, which negatively affects their QoL.^24^, ^25^, ^26^, ^27^ Compared with the general population, those with psoriasis have 40–90% more psychological comorbidities with high levels of anxiety, worry, depression, and suicidal ideation.^28^ Several factors are associated with the mental health burden in people with psoriasis, including the severity of psoriasis, duration of psoriasis, income, difficulties with sleeping, and presence of comorbidities.^27^ The Global Burden of Disease Study 2019 estimated a global prevalence of 3.8% for anxiety disorders and 3.4% for depressive disorders, with rates nearly twice (1.6 times) as high in women than men.^29^ The prevalence of anxiety disorders was high in Brazil (7.4%), the second highest rate across 204 countries, whereas the prevalence of depressive disorders was 3.8%.^29^ In this study, we reported a prevalence of 33% for anxiety or depression in people with psoriasis, which is similar to previous studies of psoriasis patients in Brazil, with rates of 36% and 19–34% documented for anxiety and depression, respectively.^30^, ^31^ This is consistent with estimates reported in other countries in psoriasis populations.^24^, ^32^, ^33^, ^34^ The high prevalence of anxiety and depression in our survey may be related to the higher proportion of female and self‐declared White^35^ participants, and it may also be associated with factors relating to social stigma and prejudice, high levels of pain and discomfort, psoriasis severity, and dissatisfaction with psoriasis treatment.

We acknowledge several limitations of our study. First, our results may be affected by selection bias; the recruitment of participants happened through email and social media platforms, and we, therefore, may have missed people with psoriasis who do not have access to these platforms, are unfamiliar with completing electronic forms, or have poor literacy proficiency; those who are more negatively affected by their psoriasis may be more willing to participate in this survey. Therefore, our data may not be representative of all patients with psoriasis. Furthermore, we did not have information on the country's geographical regions where participants resided. Psoriasis prevalence and access to care differ according to region, with higher indicators observed in the south and southeast regions.^6^, ^36^ Second, the higher proportion of White individuals in the survey than in the general Brazilian population (67.5 vs. 43.5%)^37^ may be related to ethnic differences in educational attainment and access to the internet. Inequalities in literacy persist, with illiteracy rates in Brown and Black individuals being more than double compared to those in White individuals: 8.8 and 10.1% vs. 4.3%.^38^

Additionally, a survey on the use of information and communication technologies in Brazilian households found that internet usage was higher amongst those with a higher level of education (94% of the population) compared to those with primary school level education (71%).^39^ Ethnic disparities in access to equipment, connectivity, internet usage, and connection quality were present, with better conditions observed in individuals self‐declared as White than in self‐declared Brown or Black individuals.^40^ Third, while the prevalence of psoriasis is higher in males than females,^36^ respondents were predominantly female, with a 2.7:1 female‐to‐male ratio. This may be due to the gender effect in survey participation, a social phenomenon whereby females are more likely to contribute to surveys (particularly mail and web) than males, as observed in other studies.^41^, ^42^ Fourth, as part of the EQ‐5D‐5L health‐related quality of life measure, participants were asked to self‐report symptoms of anxiety and depression. This is a screening tool and does not capture clinical diagnoses of moderate‐to‐severe anxiety and/or depression. Fifth, we captured self‐assessed psoriasis severity (saSPI) and not the professional SPI score (proSPI) assessed by healthcare professionals (including dermatologists, dermatology nurses, and primary care physicians). While severity scores obtained from the proSPI tend to be slightly lower than those obtained from the saSPI, the latter provides valuable insights into patient perceptions of their disease and its impact.^15^, ^43^ The proSPI and saSPI scores are considered complementary, and both are valid, reliable, and acceptable tools.^15^, ^43^ Despite these limitations, this study captured the life experiences of people living with psoriasis in Brazil, which extend beyond the dimensions captured in the EQ‐5D‐5L health‐related QoL and ICECAP‐A capability measures. Notably, the survey provided people with psoriasis with a platform to express their insights into the burden of the disease and the barriers they experience.

## Supporting information


**Table S1.** Demographic and clinical characteristics of survey respondents.
**Data S1.** Supplementary methods.

**Appendix S1.** Patient‐free‐text responses describing the experience of living with psoriasis.

**Appendix S2.** Use of psoriasis medications reported by survey respondents.

**Appendix S3.** Correlation between extent of psoriasis across body areas and health‐related quality of life and capability.

**Appendix S4.** Regression estimates for health‐related quality of life and capability.

## Data Availability

The data are considered “sensitive” and cannot be shared via public deposition to protect participant confidentiality. Access to the study data is available upon reasonable request from the corresponding author.
