# Supplementary material for: Experience of living with psoriasis in Brazil: a Global Psoriasis Atlas online survey
Source: Int J Dermatol. 2024 Jul 17;64(2):325–32. doi: 10.1111/ijd.17387 (PMC11771568; doi:10.1111/ijd.17387)
Supplement: Supplementary file 1 — Table S1. Demographic and clinical characteristics of survey respondents. Data S1. Supplementary methods. [file IJD-64-325-s001.docx]

**Supplementary Table 1 and other Supplementary Materials**

**Table S1.** Demographic and clinical characteristics of survey respondents

| **Characteristic** | **Number of respondents** | **Percentage of respondents** |
| --- | --- | --- |
|  | **N=563** | **%** |
| **Sex** |  |  |
| Male | 148 | 26.3 |
| Female | 414 | 73.5 |
| Prefer not to say | 1 | 0.2 |
| **Mean age (SD), years** | 42.1±12.4 | - |
| **Race** |  |  |
| White | 380 | 67.5 |
| Mixed* | 146 | 25.9 |
| Black | 30 | 5.3 |
| Asian** | 5 | 0.9 |
| Other | 2 | 0.4 |
| **Education (highest qualification)** |  |  |
| Higher university degree (PhD, Masters) | 48 | 8.5 |
| Undergraduate university degree | 287 | 51.0 |
| High School education | 194 | 34.5 |
| Primary education | 23 | 4.1 |
| Prefer not to say | 11 | 2.0 |
| **Current smoking status** |  |  |
| Smokes daily | 43 | 7.6 |
| Smokes less than daily | 23 | 4.1 |
| Does not smoke | 495 | 87.9 |
| Prefer not to say | 2 | 0.4 |
| **Past smoking status** |  |  |
| Smoked daily | 101 | 17.9 |
| Smoked less than daily | 82 | 14.6 |
| Haven't smoked | 376 | 66.8 |
| Prefer not to say | 4 | 0.7 |
| **Alcohol consumption** |  |  |
| None | 264 | 46.9 |
| Once a week | 205 | 36.4 |
| More than once a week | 86 | 15.3 |
| Daily | 8 | 1.4 |
| **Duration of psoriasis** |  |  |
| Less than 1 year | 15 | 2.7 |
| 1-2 years | 26 | 4.6 |
| 3-5 years | 62 | 11.0 |
| 6-10 years | 80 | 14.2 |
| More than 10 years | 376 | 66.8 |
| Cannot remember | 4 | 0.7 |
| **Frequency of flares in past 12 months** |  |  |
| No flare-ups | 92 | 16.3 |
| One flare-up | 259 | 46.0 |
| More than one flare-up (few/many) | 154 | 27.4 |
| Other | 58 | 10.3 |
| **Comorbidities** |  |  |
| **Presence of condition lasting or expected to last 12 months or more** |  |  |
| No | 192 | 34.1 |
| Yes | 295 | 52.4 |
| Do not know | 76 | 13.5 |
| **Mean number of comorbidities (SD)** | 2.1±1.3 | - |
| **Presence of long-term condition*** |  |  |
| Alzheimer's or other cause of dementia | 1 | 0.2 |
| Arthritis or ongoing problem with  back or joints | 170 | 30.2 |
| Blindness or partial sight | 14 | 2.5 |
| Breathing condition (e.g. asthma, COPD) | 36 | 6.4 |
| Cancer (diagnosis or treatment in  the last 5 years) | 10 | 1.8 |
| Deafness or hearing loss | 13 | 2.3 |
| Diabetes | 40 | 7.1 |
| Heart condition (e.g. angina/AF) | 11 | 2.0 |
| Hypertension | 92 | 16.3 |
| Kidney or liver disease | 46 | 8.2 |
| Mental health condition (e.g.  anxiety/depression) | 186 | 33.0 |
| Neurological condition (e.g.  epilepsy/migraine) | 30 | 5.3 |
| Schizophrenia or bipolar disorder | 6 | 1.1 |
| Stroke (affecting day-to-day life) | 1 | 0.2 |
| Ulcer or stomach disease | 28 | 5.0 |
| Other long-term condition or disability | 78 | 13.9 |
| Prefer not to say | 10 | 1.8 |
| **Measures of health** |  |  |
| Health-related quality of life score, mean±SD | 0.59±0.25 | - |
| Capability score, mean±SD | 0.71±0.21 | - |

******defined as “Brown” in Brazil census.*

*******defined as “Yellow” in Brazil census.*

**Supplementary Methods**

**Lived experience questionnaire**

The questionnaire consisted of 50 questions. Part 1 contained screening questions for eligibility.

Parts 2 and 3 collected data on the outcomes; EQ-5D-5L (EuroQoL 5-Dimension 5-Level) quality of life health status and ICECAP-A (ICEpop CAPability measure for Adults) capability measures.

The EQ-5D-5L is a questionnaire instrument designed to measure health-related quality of life and is based on five dimensions (mobility, self-care, usual activity, pain and/or discomfort, anxiety and/or depression) with five levels of severity in each dimension (“no problems”, “slight problems”, “moderate problems”, “severe problems”, and “extreme problems”). Each severity level is assigned a number with the five-number string representing the severity level of each dimension in the order in which they appear, and this describes each individual’s health profile. The numbers used for each level are: no problems=1, slight problems=2, moderate problems=3, severe problems=4 and extreme problems=5. If a person has no problems in any of the dimensions (i.e. full health state) the health profile is 11111, the health profile when there are some problems in every dimension would be 22222, and extreme problems in every dimension would be 55555. In the EQ-5D-5L there are 3,125 (5^5^) possible unique health profiles. To aggregate the EQ-5D to a single value score, weights are derived for each one of the five severity levels within each of the 5 dimensions. The weights reflect the relative importance people attach to different health problems; weights increase as severity levels increase. No problems (severity level number 1) has a weight of zero. The single score for each health profile is calculated as the deduction of the indicated severity weighted coefficient for each dimension from 1 (which indicates full health) which can range from <0 to 1. Negative scores occur if severe or extreme problems are indicated in most dimensions. A score of 1 represents ‘perfect’ health (full functional quality of life), zero represents ‘death’ and scores below zero represent ‘worse than death’.

The ICECAP-A is a measure of capability in adults which focuses on an individual’s wellbeing and capability, captured under five attributes with four response levels ranging from full capability (4) to no capability (1) in the given attribute. The attributes include; stability, attachment, independence, achievement, and enjoyment. The UK ICECAP-A tariff was used to value and consolidate the participant responses to the ICECAP-A questionnaire. The value for each individual was calculated by summing the values across the individual attributes and ranged between 0 (no capability) and 1 (full capability).

Part 4 collected data on the severity of psoriasis. Psoriasis severity was defined using the aggregated single self-assessment Simplified Psoriasis Index (SPI) severity score.^15^ This score is calculated using 2 components: the extent score and the average plaque severity score (overall state of psoriasis). For the extent score, respondents were asked to score the severity of their psoriasis in 10 separate body areas as 0 (clear or so minor that it does not bother me), 0.5 (Obvious but still leaving plenty of normal skin) or 1 (widespread involving much of the affected area). The scores across the body areas were summed to provide a single score ranging between 0 and 10 points. For the average plaque severity score, respondents were asked to give an overall average score for their condition as 0 (clear or slight redness), 1 (mild redness or scaling), 2 (definite redness), 3 (moderately severe), or 4 (very red and inflamed). The product of these 2 scores produced the SPI severity score (range 0 to 40), and this was subdivided into mild (<10), moderate (10-20), or severe (>20) psoriasis.

Other data collected included: Parts 5 and 6, psoriasis medications (prescribed medication types, other treatments, and satisfaction with their treatments) and other long-term conditions; Part 7, impact of psoriasis on work; Part 8, demographic information; and Part 9, free-text responses from participants, providing an insight into their experiences of living with psoriasis
