# Supplementary material for: Experience of living with psoriasis in Brazil: a Global Psoriasis Atlas online survey
Source: Int J Dermatol. 2024 Jul 17;64(2):325–32. doi: 10.1111/ijd.17387 (PMC11771568; doi:10.1111/ijd.17387)
Supplement: Supplementary file 2 — Appendix S1. Patient‐free‐text responses describing the experience of living with psoriasis. [file IJD-64-325-s004.docx]

**Appendix 1. Patient free-text responses describing the experience of living with psoriasis**

*Stigmatisation and prejudice*

Many respondents reported feelings of stigma, prejudice, and judgement which had an impact on their relationships, work, self-esteem, and confidence:

*“It is difficult! I've suffered a lot of prejudice, I've witnessed many exclusions, when I was a teenager I was bullied, in college I felt people were aloof or with a look of disgust.”*

*“People feel uncomfortable being close to me because of the skin lesions.”*

*“I had psoriasis lesions on my face, I believe the lesions caused me to be unselected for jobs a few times.”*

*“Aesthetically and emotionally affects. People look at it with prejudice and ‘disgust’. Those who don't know think it's transmissible.”*

*Powerlessness*

Respondents reported feeling powerless, even during periods when lesions were less apparent. Many aspects of their lives were affected including their mental health and what clothing they felt comfortable wearing:

*“Discomfort and insecurity define those who live with psoriasis.”*

*“Today it's under control, but even so I'm terrified . . . of it coming back at any moment.”*

*“Since the diagnosis and when I had my first crisis my life has changed, I have become more anxious and depressed, psoriasis rules my life even when the lesions are not so apparent. . . . I dream of the day when I will no longer be hostage to this disease.”*

*“. . . it is very difficult to live with this disease. . . . I even thought about suicide.”*

*“I feel like I’m not living, just existing.”*

*“It affects the use of clothes on a daily basis, always having to wear long pants and long-sleeved blouses to hide the marks all over the body.”*

*Education about psoriasis*

A lack of public awareness of psoriasis was highlighted by respondents which they believed led to the stigmatisation of their condition. They also highlighted limited information on treatments available to people living with psoriasis:

*“Living with psoriasis is very difficult due to people's lack of knowledge, there is a lot of prejudice . . . that ends up affecting the emotional side of people with this disease.”*

*“There is still a lack of publicity about psoriasis . . . as if it were contagious and very disgusting.”*

*". . . Psoriasis needs to be talked about to the whole world . . . we need to say that there is treatment and we can have a normal life.”*

*Difficulty obtaining appropriate care*

Patients reported barriers to accessing specialist dermatological treatment, particularly biologics:

*“[There is] difficulty in finding specialized professionals for the treatment of [psoriasis]. It has improved a lot in recent years, but it is still difficult to diagnose.”*

*“We have many people without treatment and still lost.”*

*“. . . I find it difficult to deal with doctors, we don't have many doctors willing to help with the treatment.”*

*“… it took me a long time to find a dermatologist who had the courage to recommend me [biologics]. I . . . met a dermatologist . . . she knew the treatments well, but was afraid to give the correct treatment . . . and kept saying ‘you are too young to take biological medication that will interfere with your fertility’ (I don't know where she got that from).”*

*“. . . we need to have more dermatologists specializing in diseases (psoriasis), not just focused on beauty. I was a slave for years using ointments, which are unpleasant such as: staining clothes, bedding, feeling sticky. And the efficiency for me was zero.”*
