# Supplementary material for: Experience of living with psoriasis in Brazil: a Global Psoriasis Atlas online survey
Source: Int J Dermatol. 2024 Jul 17;64(2):325–32. doi: 10.1111/ijd.17387 (PMC11771568; doi:10.1111/ijd.17387)
Supplement: Supplementary file 3 — Appendix S2. Use of psoriasis medications reported by survey respondents. [file IJD-64-325-s003.docx]

**Appendix 2. Use of psoriasis medications reported by survey respondents**

| **Characteristic** | **Number of respondents** | **Percentage of respondents** |
| --- | --- | --- |
|  | **N=563** | **%** |
| **Currently on prescribed treatment** |  |  |
| Yes, only prescribed treatment | 332 | 59.0 |
| Yes, prescribed and other non-  prescribed treatment | 81 | 14.4 |
| No, other non-prescribed treatment | 55 | 9.8 |
| No treatment | 95 | 16.9 |
| **Prescribed treatment** |  |  |
| Topical | 259 | 46.0 |
| Oral | 144 | 25.6 |
| Injectable | 176 | 31.3 |
| Light (UV) | 11 | 2.0 |
| Other | 14 | 2.5 |
| **Patient satisfied with prescribed treatment** |  |  |
| Yes | 254 | 45.1 |
| No | 77 | 13.7 |
| Not sure | 82 | 14.6 |
| Not applicable | 150 | 26.6 |
| **Currently on other non-prescribed treatment** |  |  |
| Yes | 121 | 21.5 |
| No | 442 | 78.5 |
| **Other treatment** |  |  |
| Special diet | 37 | 6.6 |
| Alternative treatment *(including*  *homeopathy and snail gel)* | 35 | 6.2 |
| Non-medical UV exposure | 2 | 0.4 |
| Sun exposure | 51 | 9.1 |
| Other | 45 | 8.0 |
| **Patient satisfied with other treatments** |  |  |
| Yes | 59 | 10.5 |
| No | 13 | 2.3 |
| Not sure | 49 | 8.7 |
| Not applicable | 442 | 78.5 |
