# Supplementary material for: Experience of living with psoriasis in Brazil: a Global Psoriasis Atlas online survey
Source: Int J Dermatol. 2024 Jul 17;64(2):325–32. doi: 10.1111/ijd.17387 (PMC11771568; doi:10.1111/ijd.17387)
Supplement: Supplementary file 4 — Appendix S3. Correlation between extent of psoriasis across body areas and health‐related quality of life and capability. [file IJD-64-325-s005.docx]

**Appendix 3. Correlation between extent of psoriasis across body areas and health-related quality of life and capability**

|  | **Quality of Life** | **Capability** |
| --- | --- | --- |
|  | Rho (r) | Rho (r) |
| **Scalp and Hairline** | -0.26* | -0.22* |
| **Face, Neck, and Ears** | -0.27* | -0.26* |
| **Arms and Armpits** | -0.20* | -0.23* |
| **Hands, Fingers, and Nails** | -0.29* | -0.26* |
| **Chest and Abdomen** | -0.27* | -0.27* |
| **Back and Shoulders** | -0.28* | -0.27* |
| **Genital and Anal Area** | -0.28* | -0.23* |
| **Buttocks and Thighs** | -0.24* | -0.25* |
| **Knees, Lower Legs, and Ankles** | -0.21* | -0.23* |
| **Feet, Toes, and Toenails** | -0.33* | -0.22* |

Strength of correlation (rho score): very weak (0 to 0.19), weak (0.20 to 0.39), moderate (0.40–0.59), strong (0.60–0.79), very strong (0.80–1).

* P<0.05
