# Supplementary material for: Experience of living with psoriasis in Brazil: a Global Psoriasis Atlas online survey
Source: Int J Dermatol. 2024 Jul 17;64(2):325–32. doi: 10.1111/ijd.17387 (PMC11771568; doi:10.1111/ijd.17387)
Supplement: Supplementary file 5 — Appendix S4. Regression estimates for health‐related quality of life and capability. [file IJD-64-325-s002.docx]

**Appendix 4. Regression estimates for health-related quality of life and capability**

|  | **Quality of Life** | | | **Capability** | | |
| --- | --- | --- | --- | --- | --- | --- |
|  | **Coefficient**  **(95% CI)** | **P-value** | **Beta value** | **Coefficient**  **(95% CI)** | **P-value** | **Beta value** |
| **saSPI score** | -0.011 (-0.013, -0.008) | <0.001 | -0.357 | -0.007 (-0.010, -0.005) | <0.001 | -0.307 |
| **Duration of Psoriasis** |  |  |  |  |  |  |
| < 1 year | 0 | - | - | 0 | - | - |
| 1 to 2 years | -0.037 (-0.179, 0.104) | 0.604 | -0.030 | 0.004 (-0.130, 0.138) | 0.954 | 0.004 |
| 3 to 5 years | -0.072 (-0.201, 0.056) | 0.270 | -0.089 | -0.026 (-0.146, 0.093) | 0.663 | -0.040 |
| 6 to 10 years | -0.028 (-0.157, 0.101) | 0.669 | -0.039 | -0.013 (-0.129, 0.103) | 0.826 | -0.022 |
| More than 10 years | -0.070 (-0.193, 0.053) | 0.266 | -0.129 | -0.018 (-0.129, 0.092) | 0.743 | -0.043 |
| I cannot remember | -0.095 (-0.255, 0.066) | 0.246 | -0.031 | -0.027 (-0.187, 0.134) | 0.745 | -0.011 |
| **Number of psoriasis flares** | -0.062 (-0.083, -0.040) | <0.001 | -0.211 | -0.034 (-0.051, -0.016) | <0.001 | -0.142 |
| **Age** | 0.001 (-0.001, 0.002) | 0.600 | 0.022 | 0.001 (-0.001, 0.002) | 0.352 | 0.040 |
| **Gender** |  |  |  |  |  |  |
| Male | 0 | - | - | 0 | - | - |
| Female | -0.077 (-0.117, -0.037) | <0.001 | -0.133 | -0.048 (-0.079, -0.017) | 0.003 | -0.103 |
| **Ethnicity** |  |  |  |  |  |  |
| White | 0 | - | - | 0 | - | - |
| Mixed | 0.040 (0.001, 0.079) | 0.043 | 0.069 | 0.011 (-0.024, 0.046) | 0.548 | 0.023 |
| Black | -0.105 (-0.178, -0.032) | 0.005 | -0.093 | -0.070 (-0.139, -0.001) | 0.047 | -0.077 |
| Asian | -0.044 (-0.282, 0.194) | 0.716 | -0.016 | 0.007 (-0.132, 0.145) | 0.925 | 0.003 |
| Other | 0.132 (-0.183, 0.448) | 0.411 | 0.031 | 0.140 (-0.133, 0.413) | 0.314 | 0.041 |
| **Comorbidity** |  |  |  |  |  |  |
| 0 | 0 | - | - | 0 | - | - |
| 1 | -0.073 (-0.115, -0.032) | 0.001 | -0.130 | -0.047 (-0.085, -0.009) | 0.016 | -0.102 |
| 2 | -0.104 (-0.153, -0.055) | <0.001 | -0.153 | -0.078 (-0.122, -0.033) | 0.001 | -0.142 |
| 3+ | -0.171 (-0.219, -0.124) | <0.001 | -0.276 | -0.116 (-0.157, -0.076) | <0.001 | -0.233 |
| **Qualification** |  |  |  |  |  |  |
| PhD/Masters | 0 | - | - | 0 | - | - |
| Full Higher Education | 0.012 (-0.039, 0.062) | 0.648 | 0.023 | -0.037 (-0.077, 0.003) | 0.066 | -0.092 |
| Full High School | -0.024 (-0.082, 0.034) | 0.410 | -0.046 | -0.063 (-0.110, -0.016) | 0.008 | -0.147 |
| Full Primary Education | -0.183 (-0.284, -0.082) | <0.001 | -0.137 | -0.146 (-0.220, -0.072) | <0.001 | -0.136 |
| Prefer not to say | -0.013 (-0.160, 0.133) | 0.858 | -0.007 | -0.135 (-0.243, -0.027) | 0.014 | -0.092 |
| **Occupation** |  |  |  |  |  |  |
| Full-time | 0 | - | - | 0 | - | - |
| Part-time | 0.007 (-0.052, 0.065) | 0.826 | 0.007 | -0.053 (-0.103, -0.003) | 0.038 | -0.065 |
| Self-employed | -0.018 (-0.067, 0.030) | 0.458 | -0.027 | -0.042 (-0.081, -0.002) | 0.039 | -0.077 |
| Retired | 0.033 (-0.036, 0.103) | 0.346 | 0.036 | 0.037 (-0.019, 0.093) | 0.196 | 0.049 |
| Unemployed | -0.109 (-0.193, -0.025) | 0.011 | -0.103 | -0.133 (-0.213, -0.054) | 0.001 | -0.156 |
| Long term sick | -0.195 (-0.357, -0.033) | 0.019 | -0.102 | -0.232 (-0.368, -0.096) | 0.001 | -0.150 |
| Homemaker | 0.046 (-0.019, 0.111) | 0.164 | 0.044 | -0.013 (-0.070, 0.044) | 0.653 | -0.015 |
| Student | 0.020 (-0.081, 0.122) | 0.691 | 0.014 | -0.047 (-0.157, 0.062) | 0.396 | -0.040 |
| Other | 0.074 (0.005, 0.144) | 0.037 | 0.051 | -0.007 (-0.090, 0.077) | 0.877 | -0.006 |

|  | **Quality of Life** | | | **Capability** | | |
| --- | --- | --- | --- | --- | --- | --- |
|  | **Coefficient**  **(95% CI)** | **P-value** | **Beta value** | **Coefficient**  **(95% CI)** | **P-value** | **Beta value** |
| **Current smoking status** |  |  |  |  |  |  |
| Not at all | 0 | - | - | 0 | - | - |
| Yes, less than daily | -0.034 (-0.112, 0.045) | 0.401 | -0.026 | 0.014 (-0.053, 0.080) | 0.689 | 0.013 |
| Yes, daily | -0.038 (-0.110, 0.034) | 0.304 | -0.040 | -0.011 (-0.066, 0.045) | 0.703 | -0.014 |
| Prefer not to say | 0.122 (-0.133, 0.377) | 0.349 | 0.029 | 0.142 (0.083, 0.201) | <0.001 | 0.042 |
| **Alcohol use** |  |  |  |  |  |  |
| Never | 0 | - | - | 0 | - | - |
| Once a week | 0.040 (0.003, 0.078) | 0.034 | 0.077 | 0.043 (0.010, 0.075) | 0.010 | 0.100 |
| More than once a week | -0.014 (-0.071, 0.043) | 0.636 | -0.020 | 0.006 (-0.036, 0.048) | 0.767 | 0.011 |
| Daily | 0.092 (-0.002, 0.185) | 0.054 | 0.043 | 0.043 (-0.048, 0.135) | 0.353 | 0.025 |
| **Prescribed Topical Treatment** |  |  |  |  |  |  |
| No | 0 | - | - | 0 | - | - |
| Yes | 0.009 (-0.026, 0.043) | 0.612 | 0.017 | 0.007 (-0.023, 0.038) | 0.635 | 0.018 |
| **Prescribed Oral Treatment** |  |  |  |  |  |  |
| No | 0 | - | - | 0 | - | - |
| Yes | -0.040 (-0.080, 0.000) | 0.051 | -0.068 | 0.026 (-0.009, 0.060) | 0.144 | 0.054 |
| **Prescribed Injection Treatment** |  |  |  |  |  |  |
| No | 0 | - | - | 0 | - | - |
| Yes | -0.047 (-0.085, -0.009) | 0.015 | -0.086 | -0.015 (-0.046, 0.016) | 0.347 | -0.034 |
| **Alternative Treatment** |  |  |  |  |  |  |
| No | 0 | - | - | 0 | - | - |
| Yes | -0.123 (-0.206, -0.041) | 0.003 | -0.118 | -0.058 (-0.126, 0.010) | 0.094 | -0.068 |
| **Sunlight** |  |  |  |  |  |  |
| No | 0 | - | - | 0 | - | - |
| Yes | -0.002 (-0.060, 0.056) | 0.949 | -0.002 | 0.053 (0.008, 0.098) | 0.020 | 0.075 |
| **Constant** | 1.014 (0.865, 1.160) | <0.001 | - | 0.973 (0.824, 1.120) | <0.001 | - |
